# Cases of Fabry Disease in Which Pathogenic Variants Are Not Detected in Parent-Child Pairs

**DOI:** 10.7759/cureus.64127

**Published:** 2024-07-09

**Authors:** Naoki Akeho, Kumiko Muta, Kenta Torigoe, Mineaki Kitamura, Takaaki Sawada, Kimitoshi Nakamura, Hiroshi Mukae, Tomoya Nishino

**Affiliations:** 1 Department of Nephrology, Izumikawa Hospital, Minamishimabara, JPN; 2 Department of Nephrology, Nagasaki University Hospital, Nagasaki, JPN; 3 Department of Pediatrics, Faculty of Life Sciences, Kumamoto University, Kumamoto, JPN; 4 Center for Clinical Genetics, Kumamoto University Hospital, Kumamoto, JPN; 5 Department of Respiratory Medicine, Nagasaki University Graduate School of Biomedical Sciences, Nagasaki, JPN

**Keywords:** enzyme replacement therapy, lyso-gb3, gla variant, α-galactosidase activity, fabry disease

## Abstract

A 15-year-old male has been experiencing fever, limb pain during exercise, and reduced sweating since childhood. During an investigation into his fever, a family history of Fabry disease was discovered, prompting a referral to our department. He was diagnosed with Fabry disease based on decreased alpha-galactosidase A (α-Gal A) activity. Concurrently, his mother was found to have experienced limb pain during fevers since childhood, and she was also diagnosed with Fabry disease based on decreased α-Gal A activity. In the genetic analysis of both individuals, the IVS1+17A>G GLA variant was identified. This variant is considered benign and not classified as a pathogenic variant. Enzyme replacement therapy has been effective in improving clinical symptoms. His sister, who has not been diagnosed with Fabry disease due to normal clinical symptoms and α-GAL A activity, also had the same variant. Among the various GLA variants, many are classified as benign rather than pathogenic. In the present cases, the possibility of other factors that cannot be identified by genetic analysis is suggested, making this case significant and worth reporting.

## Introduction

Fabry disease is an inherited X-linked disease caused by mutations in the GLA gene [1]. Due to the deficiency or reduced activity of the hydrolytic enzyme alpha-galactosidase A (α-Gal A), its substrate globotriaosylceramide accumulates in organs throughout the body, leading to organ dysfunction [1]. Current treatments for Fabry disease include enzyme replacement therapy (ERT) and chaperone therapy. Research into novel treatments such as substrate reduction therapy and gene therapy is underway, and there is increasing focus on Fabry disease, including its inclusion in newborn screening programs for early diagnosis [2,3].

Over 1000 variants of GLA have been reported to date [4]. Mutations are categorized as being associated with the classic or later-onset Fabry disease phenotype, GLA variants of unclear significance, and benign variants, respectively [5]. Benign variants are more frequent, and numerous GLA mutations have been reported as benign [6-9]. Patients diagnosed with benign variants are not considered candidates for Fabry disease-specific treatment [2].

Here, we report cases involving a mother and son diagnosed with Fabry disease based on clinical symptoms and reduced enzyme activity, despite identifying the benign variant IVS1+17 A>G. ERT was initiated, resulting in improvements in clinical symptoms. The present cases, where a pathogenic variant has not been identified, suggest the possibility of other factors that cannot be detected by genetic analysis. Therefore, it is significant and worth reporting.

## Case presentation

Case 1: 15-year-old male

Since around the age of seven, he had been experiencing burning pain accompanied by a sensation of heat in the palms and soles during fever or exercise. However, these episodes did not last long, and he had never visited a hospital for it. Additionally, he exhibited symptoms of sweating less during exercise. At age 15 in April of the year 2015, he experienced malaise and a fever of 37-38°C and developed pain in the palms and soles. Although antibiotics and acetaminophen were prescribed by a previous doctor, there was no improvement. It was discovered that there was a family history of Fabry disease, and he was referred to our department in May for further investigation and treatment. There were no notable items in the medical history.

**Figure 1:**
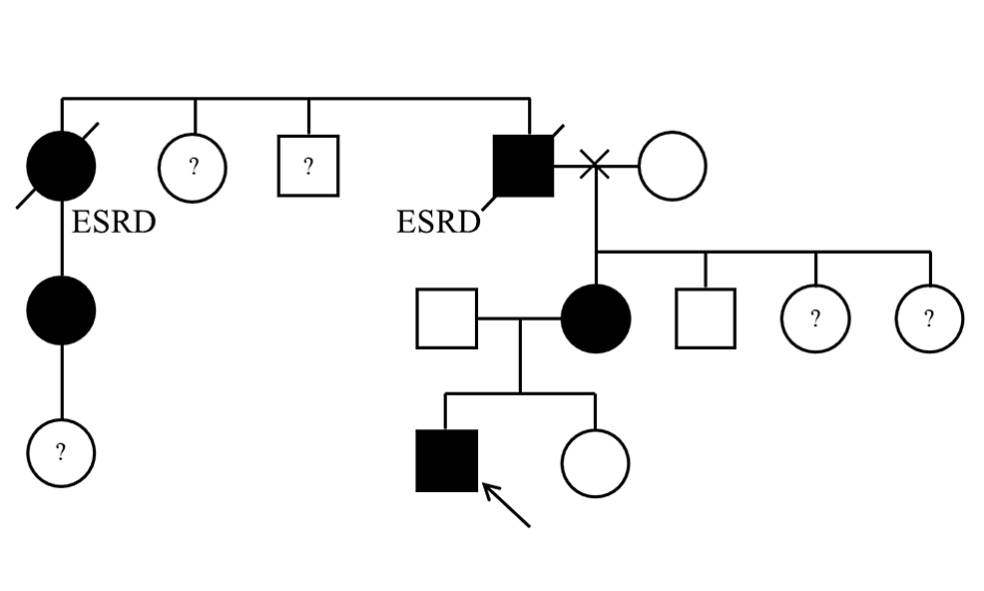
Family trees of the current case. The arrow indicates the proband. His mother was simultaneously diagnosed with Fabry disease. His grandfather and his sister were diagnosed with Fabry disease and underwent hemodialysis, and her daughter was also diagnosed with Fabry disease and has been receiving enzyme replacement therapy. A question mark indicates individuals who have either not undergone biochemical and genetic evaluations or whose testing status is unknown. ESRD: end-stage renal disease.

His grandparents were divorced, and the medical history of his grandfather's lineage, including his grandfather's, had not been communicated to him or his mother until this time. His grandfather was undergoing hemodialysis due to Fabry disease. Additionally, his grandfather's sister was diagnosed with Fabry disease and underwent hemodialysis, and her daughter was also diagnosed with Fabry disease and had been receiving ERT (Figure 1).

**Figure 2:**
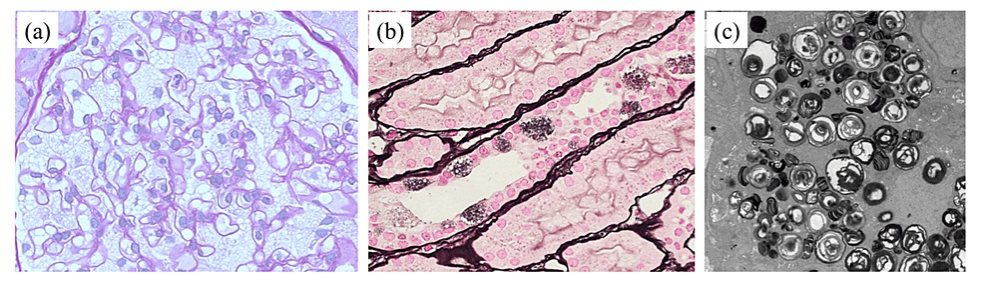
Renal pathology findings of a 15-year-old male under light microscopy and electron microscopy. (a) Glomerular epithelial cells showed vacuolar degeneration (periodic acid-Schiff staining, ×400). (b) Vacuolar degeneration was observed in some tubular epithelial cells (periodic acid–methenamine silver staining, ×400). (c) Electron microscopy revealed numerous deposits called zebra bodies in the glomerular epithelial cells (×5760).

Physical examination revealed no obvious abnormalities such as hemangioma. No abnormalities were found in the blood test results. Microalbuminuria of 99.22 mg/gCr was detected. The serum α-Gal A level was significantly reduced at 2.9 Agal U (with a cut-off value for men of <12.0), leading to a diagnosis of Fabry disease. A renal biopsy was performed to assess the extent of kidney damage. On periodic acid-Schiff (PAS) staining, vacuolar degeneration of epithelial cells was observed in all glomeruli and some tubular epithelial cells and electron microscopy revealed numerous deposits referred to as “zebra bodies” (Figure 2).

Genetic analysis revealed the IVS1+17 A>G mutation in the GLA gene in a heterozygous state. ERT and carbamazepine for pain control were initiated in October 2015. Peripheral limb pain improved, and microalbuminuria disappeared after one year. The plasma globotriaosylsphingosine (lyso-Gb3) level was not measured before treatment initiation, but it was 10.7 ng/ml in 2021.

Case 2: His mother, 38 years old

Around the age of nine years, she experienced a burning sensation accompanied by pain in the palms and soles when she had a fever about once a month. She visited a local doctor, but the cause remained unclear. At age 38 in May 2015, when her son was referred to our department with suspicion of Fabry disease, it became evident that she experienced similar symptoms. Consequently, she visited our department on the same day. She had a history of surgeries for endometriosis at the ages of 24 and 28 years. At age 38, she experienced pronounced dizziness on an empty stomach and was diagnosed with reactive hypoglycemia.

**Figure 3:**
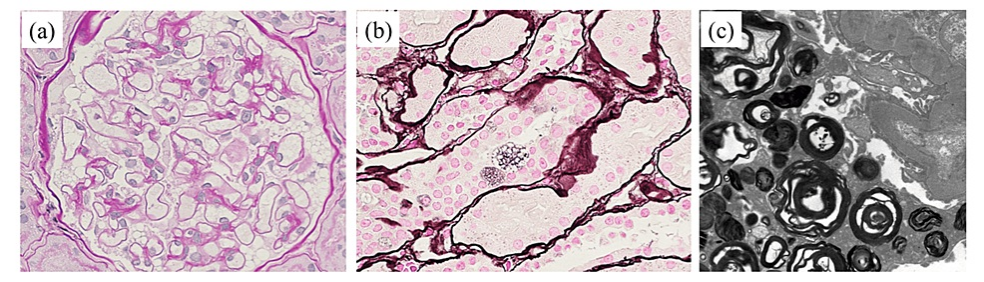
Renal pathology findings of the 38-year-old mother under light microscopy and electron microscopy. (a) Glomerular epithelial cells showed vacuolar degeneration (periodic acid Schiff staining, ×400). (b) Vacuolar degeneration was observed in some tubular epithelial cells (periodic acid–methenamine silver staining, ×400). (c) Electron microscopy revealed zebra bodies in the glomerular epithelial cells (×5760).

Physical examination revealed no obvious abnormalities such as hemangioma. Blood and urine tests showed no abnormalities. The serum α-Gal A level was significantly reduced at 9.8 Agal U (with a cut-off value for women of <20.0) and she was also diagnosed with Fabry disease. Reactive hypoglycemia was considered as a possible manifestation of autonomic nerve dysfunction due to Fabry disease. In the renal biopsy, PAS staining revealed vacuolar degeneration in nearly all glomerular epithelial cells and some tubular epithelial cells. Under electron microscopy, deposition of zebra bodies was observed (Figure 3).

Genetic analysis identified the IVS1+17 A>G mutation in the GLA gene in a heterozygous state. ERT and carbamazepine for pain control were initiated in December 2015. The pain in the limbs has been alleviated, but reactive hypoglycemia occasionally occurs. Lyso-Gb3 decreased from 25.1 ng/ml before treatment initiation to 4.42 ng/ml in 2019.

Case 3: His sister, 17 years old

Nine years after the male and his mother were diagnosed with Fabry disease, his sister visited our department seeking genetic testing for Fabry disease. She has never experienced noticeable pain in her hands or feet during childhood fever or physical activity. There were no notable physical findings. Blood and urine tests showed no abnormalities (Table 1). Urinary mulberry bodies were not detected.

**Table 1:** Laboratory findings of the 17-year-old sister. Alb, albumin; ALT, alanine aminotransferase; AST, aspartate aminotransferase; BUN, blood urea nitrogen; Cl, chloride; Cr, creatine; CRP, C-reactive protein; eGFR, estimated glomerular filtration rate; Glu, glucose; Hb, hemoglobin; K, potassium; LDH, lactate dehydrogenase; Na, sodium; P, phosphorus; Plt, platelet; TP, total protein; WBC, white blood cells.

Laboratory test	Value	Reference range
WBC (/mL)	8000	3300-8600
Hb (g/dL)	13.8	11.6-14.8
Plt (/mL)	255000	158000-348000
TP (g/dL)	7.6	6.6-8.1
Alb (g/dL)	4.0	4.1-5.1
AST (U/L)	20	13-30
ALT (U/L)	16	10-42
LDH (U/L)	227	124-222
BUN (mg/dL)	10	8-20
Cr (mg/dL)	0.76	0.46-0.79
eGFR (mL/min/1.73 m^2^)	85.84	>60
Na (mEq/L)	139	138-145
K (mEq/L)	3.8	3.6-4.8
Cl (mEq/L)	104	101-108
Ca (mg/dL)	9.3	8.8-10.1
P (mg/dL)	3.5	2.7-4.6
CRP (mg/dL)	0.01	0.00-0.14
Glu (mg/dl)	80	73-109
Microalbuminuria (mg/g·Cr)	2.26	<30
Urinary red blood cells (/HPF)	1-4	<5

The serum α-Gal A level was normal. Genetic analysis identified the IVS1+17 A>G mutation in the GLA gene in a heterozygous state. Her plasma lyso-Gb3 has not been measured. Given her family history, Fabry disease was suspected, but she lacked clinical symptoms, so she was placed under observation.

## Discussion

We encountered a case of a mother and son diagnosed with Fabry disease based on clinical symptoms and α-Gal A activity. However, the GLA variant was considered benign, and no pathogenic variant could be identified.

Fabry disease is caused by a deficiency or dysfunction of α-Gal A level due to mutations in the GLA gene and presents a wide range of symptoms. Early symptoms include skin abnormalities such as angiokeratomas, corneal opacities (corneal verticillata), pain due to autonomic nervous system involvement (acroparesthesia), and abnormal sweating (anhidrosis or hypohidrosis). As the disease progresses, it can lead to severe complications, such as renal failure, cardiomyopathy, and cerebrovascular events [2]. Fabry disease presents with classical and late-onset phenotypes in male patients [1]. Plasma lyso-Gb3 is widely used as a marker associated with disease progression and severity of the condition [10]. Early diagnosis and treatment are crucial, as prognosis can be improved with ERT and chaperone therapy. The son in the present case is considered to have the classical type, and the mother also reported symptoms since childhood. ERT has been successful in improving their clinical symptoms and the mother's plasma lyso-Gb3 levels decreased after the start of ERT. The pre-treatment plasma lyso-Gb3 levels of the son were not measured, but six years after starting treatment, the reported values are significantly lower than those typically seen in classic male cases, suggesting a favorable prognosis [10]. His sister is suspected of having Fabry disease due to family history, but she has not been diagnosed with it because her clinical symptoms and α-Gal A activity are normal.

If a specific variant among GLA variants is frequently observed in control populations of healthy individuals and shown not to be associated with the incidence of Fabry disease, it may be classified as benign [5]. Variants such as p.D313Y, p.E66Q, and p.R118C in the GLA gene have been reported to have a prevalence in the general population that is equal to or higher than that in clinical or registry populations [6-9]. At the time the present cases were diagnosed with Fabry disease, the IVS1+17A>G mutation had not been reported elsewhere and was classified as having unknown clinical significance. However, subsequent reports of this variant have accumulated, and there are now multiple entries in ClinVar (https://www.ncbi.nlm.nih.gov/clinvar/?term=c.194%2B17A%3EG), all of which classify this variant as benign. Therefore, this mutation is considered non-pathogenic in the present cases. Several relatives of the present cases have been diagnosed with Fabry disease, but the results of their genetic analysis are not known. The clinical symptoms and reduced enzyme activity in the present cases may be attributed to other pathogenic mutations or factors affecting the GLA gene. For example, mutations in enhancer regions of GLA, or epigenetics might be responsible for the onset of Fabry disease, which cannot be detected through gene analysis in general. Recently, a study reported an association between GH0XJ101390, an enhancer of the RPL36 gene located within the X chromosome genomic region where the GLA gene resides, and the expression of the GLA gene in vitro [11]. Epigenetics involvement in Fabry disease includes the crucial role of DNA methylation in X chromosome inactivation, as well as reports indicating correlations between the methylation status of the GLA gene promoter region in female Fabry disease patients and disease severity [12]. Many aspects of the above remain unclear, and further insights are awaited. If the sister in this case were believed to have developed Fabry disease due to these factors, there is debate over the timing of ERT initiation in asymptomatic female patients [5]. Her plasma lyso-Gb3 has not been measured, but if it is found to be elevated or if clinical symptoms and organ damage appear, initiating ERT might need to be considered.

## Conclusions

These cases of Fabry disease did not show detectable pathogenic variants in parent-child pairs. ERT has been effective in improving their clinical symptoms and is necessary to continue for the prognosis improvement of organ damage. The possibility of other mutations or factors that cannot be identified by genetic analysis is suggested, indicating the need for further research. Due to suspicion of Fabry disease in his sister as well, we will carefully monitor for any signs of clinical symptoms or organ dysfunction in the future.
